# User Experience Evaluation in Shared Interactive Virtual Reality

**DOI:** 10.1089/cyber.2022.0261

**Published:** 2023-04-14

**Authors:** Shady Guertin-Lahoud, Constantinos K. Coursaris, Sylvain Sénécal, Pierre-Majorique Léger

**Affiliations:** ^1^ Department of Information Technologies, HEC Montréal, Montréal, Quebec, Canada.; ^2^ Department of Marketing, HEC Montréal, Montréal, Quebec, Canada.

**Keywords:** virtual reality, multiuser virtual environments, immersive user experience, copresence, interactivity, emotional arousal

## Abstract

Virtual reality (VR) has served the entertainment industry all the way to world-leading museums in delivering engaging experiences through multisensory virtual environments (VEs). Today, the rise of the Metaverse fuels a growing interest in leveraging this technology, bringing along an emerging need to better understand the way different dimensions of VEs, namely social and interactive, impact overall user experience (UX). This between-subject exploratory field study investigates differences in the perceived and lived experience of 28 participants engaging, either individually or in dyads, in a VR experience comprising different levels of interactivity, i.e., passive or active. A mixed methods approach combining conventional UX measures, i.e., psychometric surveys and user interviews, as well as psychophysiological measures, i.e., wearable bio- and motion sensors, allowed for a comprehensive assessment of users' immersive and affective experiences. Results pertaining to the social dimension of the experience reveal that shared VR elicits significantly more positive affect, whereas presence, immersion, flow, and state anxiety are unaffected by the copresence of a real-world partner. Results pertaining to the interactive dimension of the experience suggest that the interactivity afforded by the VE moderates the effect of copresence on users' adaptive immersion and arousal. These results support that VR can be shared with a real-world partner not only without hindering the immersive experience, but also by enhancing positive affect. Hence, in addition to offering methodological directions for future VR field research, this study provides interesting practical insights into guiding VR developers toward optimal multiuser virtual environments.

## Introduction

Fuelled by technological progress, society has entered the “experience age,” constantly seeking novelty. Virtual reality (VR), a next-generation technology enabling immersive experiences through vivid three-dimensional virtual environments (VEs), successfully aligns with this social eagerness.^1^ Hence, VR is increasingly used in the arts & entertainment sector, namely in museum contexts to enhance user experience (UX) by reinventing content delivery and boosting crowd engagement.^2^ This unique potential places VR under the spotlight, which is shining brighter since the rise of the Metaverse.

Best known for merging physical and digital realities within a multiuser virtual environment (MUVE), the Metaverse is a transformative technology that not only allows for seamless interactions with virtual objects, but also promotes social networked and embodied interactions.^3,4^ With the aim to be adopted by the general public, the Metaverse brings about a growing need to better understand UX in VR, specifically in real-life contexts of use. To our knowledge, VR research carried out in the field is scarce and typically focuses on educational, rather than entertainment, purposes.^5^ Thus, this study conveys important contributions by taking place outside a controlled laboratory environment, in a multimedia entertainment center, and by focusing on social and interactive dimensions of VR.

In VR, there seems to be an emerging duality between creating a social yet optimally immersive experience. By definition, VR is a technology that requires users to block all external distractions to plunge into a virtual world, making it particularly vulnerable to events, for example, social interactions, occurring in the real world.^6,7^ Relatedly, an essential tenet of social psychology is that one's behavior is influenced by different social contexts.^8^ However, although VR developers are investing efforts in creating shared VEs, relatively little research has empirically evaluated the effect of world-based social interactions during immersive experiences.^9,10^

In addition to enabling social interactions, optimal VR experiences are also those affording seamless interactions with virtual objects and the VE itself.^4^ Previous research, however, does not offer a clear consensus pertaining to a VE's optimal level of interactivity. In fact, some researchers support that high or low interactivity levels should be prioritized over moderate interactivity levels^11^; others argue that a VE's interactivity needs to be properly aligned with the experience's purpose.^12^ Together, these current gaps fuel the following research questions.

***RQ1: How does the copresence of a real-world partner in a shared virtual environment influence the user experience in VR?***
***RQ2: How does the interactivity of the virtual environment influence the user experience in VR?***

### Individual versus shared VR

#### Immersive UX

*Presence* is defined as the subjective sense of “being there” in an environment, even when one is physically situated in another.^13^ In VR, presence is contingent upon the ability to block irrelevant real-world stimuli and focus on actions occurring in the VE.^13^ In MUVEs, presence is extended to *copresence*, i.e., “being there *together*.”^14^ Relatedly, *immersion* is based on the technology's affordances including image resolution or sound quality.^15^

When presence and immersion are optimal, *flow*, i.e., a mental state of absolute absorption and involvement, is more likely to emerge.^16^ Altogether, considering immersive UX being contingent on external stimuli, and aligned with previous research supporting that active distractions decrease social presence,^17^ we hypothesized that the copresence of a real-world partner would hinder the immersive user experience by decreasing a user's presence (H1a), adaptive immersion (H1b), sensory immersion (H1c), and flow (H1d).

#### Affective UX

*Positive affect* refers to the combination of pleasurable emotions (i.e., feeling content and happy).^18^ The other end of the emotional spectrum comprises negative affect including *state anxiety*, i.e., a transitory anxious emotional state elicited by the activity at hand,^19^ which could arise from experiencing a novel VE.

However, past studies building upon social facilitation, a well-established theory supporting that social contact leads to emotional happiness,^20^ showed that sharing an experience increases feelings of reward, pleasantness, and enjoyment,^21,22^ therefore, serving as a successful mechanism in coping with various life stressors, even that of physical pain.^23,24^ Hence, we hypothesized that the copresence of a real-world partner would enhance the affective user experience by leading to greater positive affect (H2a) and lower state anxiety (H2b).

#### Lived UX

*Emotional arousal,* measured through electrodermal activity (EDA), refers to the intensity of an emotional experience.^25^ High EDA is related to curiosity/anxiety, whereas low EDA is related to relaxation/boredom.^26^ Greater emotional arousal is also inferred from higher heart rates (HRs) measured through an electrocardiogram (ECG).^27^ Another measure of lived experience consists of *exploration behavior*, measured through users' motion in their physical environment. Previous research found that both emotional arousal and exploration behavior fluctuate per the social context of an experience. For instance, solo participants walk, i.e., explore, less during spatial VR tasks than those in a collaborative colocated condition.^28^

Combining extant findings and theories that sharing an experience serves as a stress coping mechanism,^23,24^ we hypothesized that the copresence of a real-world partner would be associated with lower emotional arousal, i.e., decreased electrodermal activity (H3a) and heart rate (H3b), but higher exploration behavior (H4).

### Passive versus active VR

*Interactivity*, the degree through which the content of a VE can be influenced by users' actions, translates into the passive–active spectrum of a VR experience.^29^ Although passive experiences do not afford users to interact with the VE, active experiences do.^30^ Given that active VR replicates more accurately real-life experiences than passive VR, previous research reported higher presence, more positive affect, and intensified emotional reactions during the former.^31–34^

**FIG. 1.**
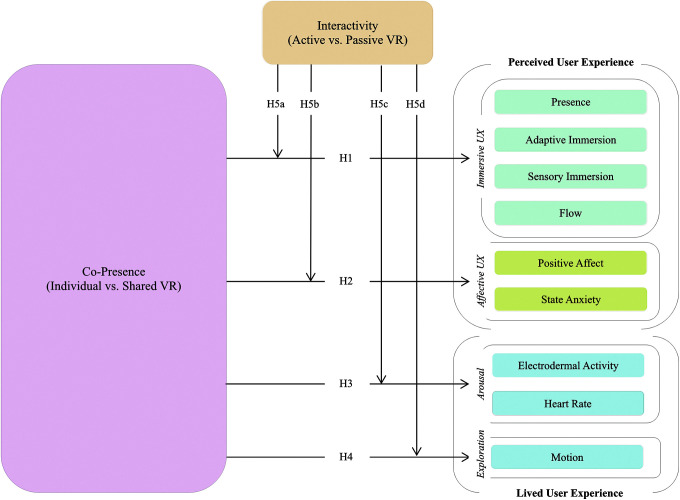
Research model.

Thus, considering that interactivity is contingent upon the VE's affordances with surrounding entities, for example, objects or humans, we hypothesized that an interaction effect between copresence and a VE's interactivity would be negatively associated with immersive UX (H5a) and users' emotional arousal (H5c), but positively associated with affective UX (H5b) and exploration behavior (H5d), as shown in Figure 1.

## Materials and Methods

### Sample

This study was completed by a convenience sample of 28 participants (*F* = 12, *M* = 16), aged 20–34 years old (*M* = 24.71, *SD* = 3.17), recruited through a word-of-mouth snowball sampling process per the selection criteria detailed in Table 3. Based on a screener survey, selected participants reported a normal or corrected-to-normal vision and no history of psychiatric or neurological disorders. Participants were also screened for motion sickness propensity, but all were retained.

The study was approved by the research ethics board of the authors' institution, with participants' written consent obtained at the time of the study (Certificate No. 2022-4458). The minority, i.e., nine participants, were novice VR users, whereas the remainder had used it at least once. Participants were provided free entry to the VR experience, valued at CA$45, as compensation.

### Experimental design

This field study was undertaken at a multimedia entertainment center located in Montreal, Canada, which hosted the VR experience that served as the stimulus in this study. It employed a between-subject design manipulating the social context of the experience. At the time of enrollment, participants signed up individually or in pairs with a friend or romantic partner. Accordingly, participants were either assigned to the solo group, composed of 12 participants (*F* = 4, *M* = 8) who underwent the experience on their own (among strangers), or the duo group, composed of 16 participants (i.e., 8 duos) (*F* = 8, *M* = 8), who underwent the experience with their real-world partner (among strangers).

### Virtual environment

**FIG. 2.**
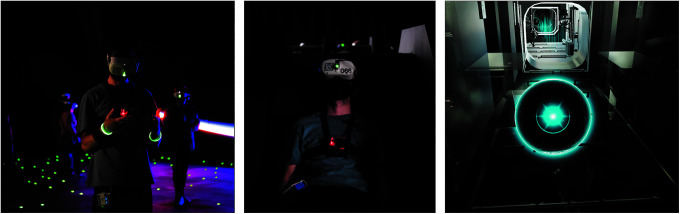
Participant undergoing the active standing phase of the experience (*left*), followed by the passive seated phase of the experience (*middle*). Virtual representation of the ISS featuring a virtual luminous sphere (*right*). ISS, International Space Station.

The VR experience was powered through the Oculus Quest 2 head-mounted display (HMD; Facebook, Inc., Menlo Park, CA). A state-of-the-art color-coded tracking system allowed users to portray their body virtually, recognize their partner among strangers, monitor others' locations, and interact with the VE using their hands, free of controllers. The VR experience, taking users in space aboard the International Space Station (ISS), comprised two phases: a 30-minute active standing phase followed by an 8-minute passive seated phase (Fig. 2). In the first phase, participants were brought to walk freely through a 3D modelized ISS representation and interact with luminous spheres.

Upon touching, each sphere launched a 360° video showcasing astronauts undergoing daily activities; participants could explore this VE through desired head movements and on-site body rotations. In the second phase, participants were virtually guided to a physical chair to watch an unnarrated rotation around planet Earth from the ISS cupola. Participants' HMD states, including potential technical issues (e.g., low battery and erroneous tracking) and their progression through the VR experience were monitored in real time on the moderator's tablet.

### Procedure

**FIG. 3.**
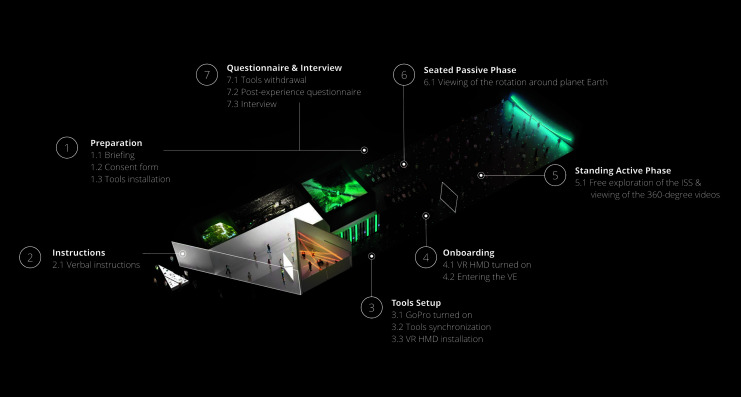
Experimental procedure detailed along the exhibition room plan.

Participants were first welcomed on site and directed to a preparation room (Fig. 3). Once briefed on the tools and general format of the experiment, their consent was obtained. Participants were instructed to behave as naturally as possible during the VR experience; verbal and physical interactions were allowed among dyads. Participants changed into the Hexoskin Smart Garment (Carré Technologies, Inc., Montreal, Canada), to which a GoPro was fixed (Hero 4; Woodman Labs, Inc., San Mateo, CA), and fitted with EDA sensors in the palm of their nondominant hand (Cobalt System; Tech3Lab, Montreal, Canada).

Participants were then guided toward the exhibition entrance where instructions on virtual space navigation were provided. The GoPro was turned on, tools were synchronized, the HMD was installed, and participants began the experience. The moderator withdrew, but stayed in the room to overview potential technical issues. At the end, participants were brought back to the preparation room to conclude with a postexperience questionnaire and an individual semistructured interview.

### Measures and tools

#### Psychometric self-reports

To assess perceived experience, a self-report postexperience questionnaire comprised previously validated adapted measures of copresence,^35^ presence,^36^ immersion (assessed through adaptive immersion^37^ and sensory immersion^38^), flow,^38^ positive affect,^38^ and state anxiety,^19^ all of which using a 7-point Likert scale (Table 3). The internal consistency of scales ranged from acceptable (α = 0.706) to excellent (α = 0.937) (Table 3).

#### Psychophysiological and motion sensors

To assess lived experience, exploration behavior was measured through a noninvasive 64 Hz accelerometer embedded in the Hexoskin. A record of unexpected events or technical issues was provided by the GoPro. Emotional arousal was inferred from HR recorded by a 256 Hz ECG embedded in the Hexoskin, and EDA measured through disposable Ag/AgCl sensors.

### Data preprocessing

Data synchronization and behavioral/stimuli coding was performed using Observer XT (Noldus, Wageningen, the Netherlands). GoPro recordings were visually inspected such that atypical events, for example, technical problems or strangers' physical/verbal interruptions, were removed from the data.

### Statistical analyses

Given the moderate sample size and a lack of normality observed in the psychophysiological data, differences across social conditions with regard to dependent variables were examined using nonparametric Wilcoxon sum rank one-tailed tests. In addition, the interaction between copresence and a VE's interactivity on dependent variables was tested using linear and logistic regressions with random intercept. Statistical analyses were performed using SPSS Statistics (Version 28.0; IBM Corp., Armonk, NY), with a threshold for significance set at *p* < 0.05.

## Results

### Manipulation check

To ensure that the social context was manipulated as desired, copresence mean scores were compared between social conditions. Results confirmed that participants in dyads (*M* = 5.509, *SD* = 0.71) experienced significantly higher levels of copresence than solos (*M* = 4.536, *SD* = 0.95, *z* = 2.722, *p* = 0.005).

### Perceived UX results

Results outlined in Table 1 show that no significant difference in users' immersive experience emerged between social conditions, thus H1 is not supported. In contrast, with regard to users' affective experience, positive affect was significantly higher for dyads than solos (*p* = 0.010), whereas state anxiety did not significantly differ between social conditions. Accordingly, H2a is supported while H2b is not, offering partial support for H2 (Table 2). Furthermore, results showed that the interactivity afforded by a VE moderates the relationship between copresence and adaptive immersion (*p* < 0.0001), therefore, partially supporting H5a (Table 3).

**Table 1.** Copresence Effects on Perceived and Lived User Experience

	Presence					Adaptive immersion					Sensory immersion				
	M	Median	SD	Z	*p*	M	Median	SD	Z	*p*	M	Median	SD	Z	*p*
Solo	4.625	4.500	1.051	0.580	0.283	5.592	5.750	0.704	−0.116	0.454	5.962	6.333	0.859	−0.743	0.232
Duo	4.316	4.500	1.357			5.634	5.875	0.700			6.307	6.333	0.707		

	Flow					Positive affect					State anxiety				
	M	Median	SD	Z	*p*	M	Median	SD	Z	*p*	M	Median	SD	Z	*p*
Solo	5.471	5.600	1.305	−1.045	0.153	5.608	5.800	1.086	−2.491	0.010	2.016	1.800	0.859	−0.209	0.418
Duo	5.929	6.200	1.207			6.489	6.800	0.718			2.078	1.600	1.005		

	HR					EDA					Motion				
	M	Median	SD	Z	*p*	M	Median	SD	Z	*p*	M	Median	SD	Z	*p*
Solo	73.096	78.199	17.976	−0.489	0.315	1.562	1.409	0.672	−0.651	0.261	0.014	0.012	0.013	−1.414	0.085
Duo	73.501	80.300	27.306			1.489	1.472	0.232			0.016	0.014	0.012		

Results from Wilcoxon sum rank one-tailed tests investigating differences across social grouping conditions with regard to perceived experience (i.e., presence, adaptative immersion, sensory immersion, flow, state anxiety, and positive affect) and lived experience (i.e., EDA, HR, and motion).

EDA, electrodermal activity; HR, heart rate.

**Table 2.** Table of Hypotheses Testing for RQ1 and RQ2

H	From	Directionality	To	Z or b	SE	DF	*t*	*p*	Status	Lower	Upper
1a	CP	↓	Presence	*Z* = 0.5803				0.567	Not supported		
1b	CP	↓	Adaptive immersion	*Z* = −0.1161				0.909	Not supported		
1c	CP	↓	Sensory immersion	*Z* = −0.7429				0.464	Not supported		
1d	CP	↓	Flow	*Z* = −1.0445				0.306	Not supported		
2a	CP	↑	Positive affect	*Z* = −2.4905				0.019	Supported		
2b	CP	↓	State anxiety	*Z* = −0.2089				0.836	Not supported		
3a	CP	↓	EDA	*Z* = −0.6512				0.522	Not supported		
3b	CP	↓	HR	*Z* = −0.4886				0.629	Not supported		
4	CP	↑	Exploration	*Z* = −1.4144				0.170	Not supported		
5a	CP × interactivity	↓	Presence	*b* = 0.054	0.113	300	0.480	0.631	Not supported	−0.167	0.276
	CP × interactivity	↓	Adaptive immersion	*b* = 0.0549	0.105	300	5.230	<0.0001	Supported	0.343	0.756
	CP × interactivity	↓	Sensory immersion	*b* = 19.1051	13.353	300	1.430	0.154	Not supported	−7.172	45.382
	CP × interactivity	↓	Flow	*b* = 2.1285	1.785	300	1.190	0.234	Not supported	−1.385	5.642
5b	CP × interactivity	↑	Positive affect	*b* = 3.5417	2.090	300	1.690	0.091	Not supported	−0.571	7.655
	CP × interactivity	↓	State anxiety	*b* = −1098.1	0.000	300	M	<0.0001	Not supported		
5c	CP × interactivity	↓	EDA	*b* = −0.2171	0.079	260	−2.760	0.006	Supported	−0.372	−0.062
	CP × interactivity	↓	HR	*b* = −3.6042	4.124	299	−0.870	0.383	Not supported	−11.720	4.511
5d	CP × interactivity	↑	Exploration	*b* = −0.00179	0.004	299	−0.450	0.656	Not supported	−0.010	0.006

↑ Enhancing effect; ↓ dampening effect.

CP, copresence.

**Table 3.** Questionnaires and Scales Internal Consistency

Questionnaire	Construct	Scale or criteria	Reference	No. of items	Likert scale	α
Post-experience self-report	Co-presence	Poeschl and Doering's Co-presence subscale	Poeschl and Doering^35^	7	1: Not at all 7: Completely	0.754
	Presence	Slater–Usoh–Steed presence questionnaire	Usoh et al.^36^	6	1: Not feeling there at all 7: Feeling as present as in the real world	0.831
	Adaptive immersion	Presence questionnaire, Adaptation/immersion subscale	Witmer et al.^37^	8	1: Not at all 7: Completely	0.747
	Sensory immersion	Game experience questionnaire, Sensory/imaginative immersion component	IJsselsteijn et al.^38^	6	1: Not at all 7: Extremely	0.895
	Flow	Game experience questionnaire, Flow component	IJsselsteijn et al.^38^	5	1: Not at all 7: Extremely	0.886
	Positive affect	Game experience questionnaire, Positive affect component	IJsselsteijn et al.^38^	5	1: Not at all 7: Extremely	0.937
	State anxiety	STAIS-5	Zsido et al.^19^	5	1: Not at all 7: Very much so	0.706
Screener	Cybersickness	MSSQ-short	Golding, 2006^44^	18	1: Not applicable 5: Frequently felt sick	0.875
	Inclusion criteria	Be older than 18 years old				
		Understand spoken/written French at an advanced level				
		Understand spoken/written English at an advanced level				
		Hold a valid COVID-19 vaccination passport				
		Be able to use a VR headset without feeling nauseous				
		Be able to use a VR headset without prescription glasses				
	Exclusion criteria	Have previously attended this specific VR experience				
		Have skin allergies or particular skin sensitivities				
		Have a neurological or psychiatric diagnosis				
		Have a pacemaker				
		Have a diagnosed health problem				
		Suffering or having suffered from epilepsy				

VR, virtual reality.

**FIG. 4.**
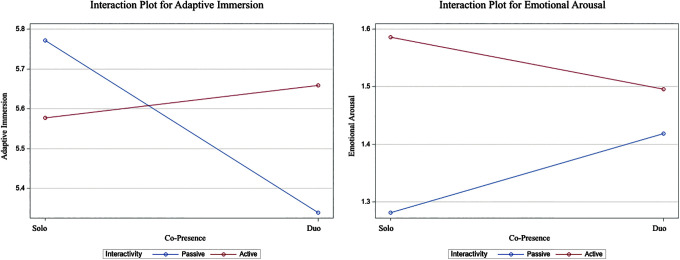
Interaction plots of the interaction effect between copresence and a VE's interactivity on measures of adaptive immersion and electrodermal activity. VE, virtual environment.

That is, the effect of sharing a VR experience with a real-world partner on adaptive immersion depends on whether that experience is of passive or active nature. Specifically, solos reported higher adaptive immersion during passive VR, whereas dyads reported higher adaptive immersion during active VR (Fig. 4).

### Lived UX results

Results outlined in Table 1 show no significant difference in participants' emotional arousal and exploration behavior across social conditions. Hence, H3 and H4 are not supported (Table 2). In contrast, results showed that copresence effect on EDA depends on the interactivity of the VR experience (*p* = 0.006), therefore, partially supporting H5c (Table 3). Specifically, EDA was higher during active than during passive VR for solo participants, whereas this significant effect was not detected in dyads (Fig. 4).

### Qualitative results

#### Shared experience elicits greater positive affect through avatar tracking and gamification

The majority of the sample, i.e., 20 of 28 participants, reported they preferred or would have preferred (if in the solo group) performing the experience in dyads (Table 4). The main justification being the physical proximity afforded by a real-world partner, as participants “would try to avoid other strangers, simply touching or following [their] companion made [the overall experience] more pleasant” (P19). In addition, what participants preferred was “to see the avatars walking around while being connected with a friend” (P05).

**Table 4.** Qualitative Insights Summary

Qualitative insight	Solo frequency	Duo frequency	Total frequency
Preference for having shared the experience	6/12 participants	14/16 participants	20/28 participants
Feeling more present during the active VR phase	6/12 participants	9/16 participants	16/28 participants
Feeling more excited and awake postexperience	4/12 participants	8/16 participants	12/28 participants

Frequency of each qualitative insight per social grouping condition.

Thus, positive affect was elevated through the VE's sophisticated tracking system, becoming more salient in the shared condition. In addition, the use of gamification, enabled through interactions between partners' avatars, seemed to enhance the positive affect experienced by dyads: “being accompanied, felt a bit more like a game” (P27).

#### Active VR elicits greater presence through interactivity

The majority of the sample, i.e., 16 of 28 participants, reported feeling more present during the active phase, a feeling repeatedly attributed to the greater interactivity afforded by the VE (Table 4). Oppositely, “as soon as [they] sat down, no interaction allowed [them] to stay focused on what [they were] experiencing in VR, so [their] attention was automatically turned to their own thoughts” (P12). This suggests that interacting with the VE helps in remaining connected to the on-hand experience, therefore, optimizing users' presence.

#### The overall experience elicits positive states of mind, but can appear physically strenuous

When queried about their overall state postexperience, 12 of 28 participants reported feeling more excited and awake than before beginning the experience (Table 4). Despite this seemingly general positive trend, some important nuances were raised by participants feeling “a little more excited and awake (postexperience), [but] ‘a little physically drained’ (P03) as “the standing phase asked for more energy, (…) [specifically] having to always pay attention to collisions” (P04).

## Discussion

### Contributions and implications

This field study investigated the effects of social and interactive dimensions of VR on UX. The most relevant implications are addressed next through theoretical, practical, and methodological lenses.

Revisiting RQ1, and the effect of the social context on affective UX, results showed that shared, rather than individual, active VR elicits greater positive affect (H2a). Not only does this finding support existing social theories, namely Zajonc's social facilitation theory,^20^ but also prior research on other immersive media showing that shared gaming experiences elicit greater enjoyment and fun.^22,39^

Although results pertaining to immersive UX offered an opposite directionality than the one hypothesized, i.e., no significant difference in users' presence, immersion, and flow between social conditions, qualitative results help in explaining this unexpected trend; per user interviews, it seems like the sophisticated tracking system was successful in integrating real-world partners to a point where their presence became a seamless part of the VE, which is aligned with previous research showing that presence is facilitated when VR elicits genuine emotional and behavioral responses.^40^

Considering the growing eagerness to develop social MUVEs for the Metaverse,^41^ this is a notable practical implication for VR developers by supporting that VR, contingent upon well-designed real-time tracking, can be successfully shared, such that the introduction of a real-world partner is not a threat to the elicited immersive UX.

Revisiting RQ2, and the effect of a VE's interactivity, results suggest that the interactivity afforded by the VE moderates the effects of copresence on adaptive immersion and EDA. Specifically, adaptive immersion was greater during passive VR for solos while it was greater in active VR for dyads. This implies that adaptive immersion is reinforced through greater VE interactivity in contexts of shared VR, thus serving as a motivation for VR developers to prioritize a VE's interactivity when designing shared experiences.

From a methodological point of view, our successful mixed methods approach combining a variety of psychophysiological measures through advanced wearable technology appears as a notable contribution toward the ecological evaluation of VR experiences for a variety of real-life entertainment applications.

### Limitations and future research

Load sharing, measured through emotional arousal, is mediated by physical contact.^42^ In this study, however, although dyads were made of friends or romantic partners, the strength of their relationship, along with the extent to which they talked, touched, or remained physically close during their experience, was not controlled for. Although this is a limitation to our results, it also sets path for future research to further investigate the effects of dyadic relationships on lived and perceived UX.

## Conclusion

Overall, this field study sheds light onto the social and interactive dimensions of VR, by evaluating immersive and affective experiences. It successfully leverages a mixed methods approach in taking UX VR research out of controlled laboratory settings, into the field. Hence, ecologically valid results provide confidence that VR can be shared with a real-world partner without diminishing the immersive experience, and that immersion is partly reinforced through a VE's interactivity during shared VR.

This conclusion unlocks a variety of virtual social opportunities, and it stresses the importance for VR developers to focus on creating reliable virtual transposition of real-world entities through sophisticated avatar tracking systems for instance. Our results suggest a particularly promising future for the Metaverse, by pointing in a direction that agrees with Palmer Luckey, founder of the Oculus, claiming that “VR is a way to escape the real world into something more fantastic. It has the potential to be the most social technology of all time.”^43^
